# Evaluating the Response of AI-Based Large Language Models to Common Patient Concerns About Endodontic Root Canal Treatment: A Comparative Performance Analysis

**DOI:** 10.3390/jcm14217482

**Published:** 2025-10-22

**Authors:** Busra Demir Cicek, Orhan Cicek

**Affiliations:** 1 Zonguldak Oral and Dental Health Center, Zonguldak Provincial Health Directorate, Zonguldak 67020, Türkiye; busra.dmr67@gmail.com; 2 Department of Orthodontics, Faculty of Dentistry, Zonguldak Bulent Ecevit University, Zonguldak 67600, Türkiye

**Keywords:** artificial intelligence, large language models, root canal treatment, patient education as topic, health literacy, endodontics, frequently asked questions (FAQs)

## Abstract

**Objectives:** The aim of this study was to compare the responses of large language models (LLMs)—DeepSeek V3, GPT 5, and Gemini 2.5 Flash—to patients’ frequently asked questions (FAQs) regarding root canal treatment in terms of accuracy and comprehensiveness, and to assess the potential roles of these models in patient education and health literacy. **Methods:** A total of 37 open-ended FAQs, compiled from American Association of Endodontists (AAE) patient education materials and online resources, were presented to three LLMs. Responses were evaluated by expert clinicians on a 5-point Likert scale for accuracy and comprehensiveness. Inter-rater and test–retest reliability were assessed using intraclass correlation coefficients (ICCs). Differences among models were analyzed with the Kruskal–Wallis H test, followed by pairwise Mann–Whitney U tests with effect sizes (Cliff’s delta, δ). A *p*-value < 0.05 was considered statistically significant. **Results:** Inter-rater agreement was excellent, with ICCs of 0.92 for accuracy and 0.91 for comprehensiveness. Test–retest reliability also demonstrated high consistency (ICCs of 0.90 for accuracy and 0.89 for comprehensiveness). DeepSeek V3 achieved the highest scores, with a mean accuracy of 4.81 ± 0.39 and a mean comprehensiveness of 4.78 ± 0.41, demonstrating statistically superior performance compared to GPT 5 (accuracy 4.0 ± 0.0; comprehensiveness 4.05 ± 0.4; *p* < 0.05, δ = 0.81 for accuracy, δ = 0.69 for comprehensiveness) and Gemini 2.5 Flash (accuracy 3.83 ± 0.68; comprehensiveness 3.81 ± 0.7; *p* < 0.05, δ = 0.71 for accuracy, δ = 0.70 for comprehensiveness). No significant difference was observed between GPT 5 and Gemini 2.5 Flash for either accuracy (*p* = 0.109, δ = 0.16) or comprehensiveness (*p* = 0.058, δ = 0.21). **Conclusions:** LLMs, such as DeepSeek V3, which can provide satisfactory responses to FAQs may serve as valuable supportive tools in patient education and health literacy; however, expert clinician oversight remains essential in clinical decision-making and treatment planning. When used appropriately, LLMs can enhance patient awareness and support satisfaction throughout the root canal treatment.

## 1. Introduction

Artificial intelligence (AI), introduced in the 1950s, refers to the development of machines capable of performing tasks traditionally carried out by humans [1]. AI uses algorithms, mainly through machine learning, to simulate intelligent behavior and support data collection, analysis, and imaging [2,3]. In dentistry, the use of AI has rapidly expanded in recent years, enhancing clinical decision-making and patient care while reducing errors [1]. Large language models (LLMs) possess the ability to understand human language and generate new content, enabling them to answer questions, translate texts, and produce articles based on extensive training data [4]. In dentistry, LLMs help in diagnosis by analyzing patient records and imaging, and guide treatment planning with evidence-based recommendations [5]. As these models continue to advance, they hold significant potential to transform healthcare delivery and improve patient outcomes.

Endodontic diseases, including pulpitis, periapical periodontitis, and dental trauma, have a high global prevalence and cause significant pain and discomfort for patients [5]. Accurate diagnosis and timely treatment are critical for tooth preservation, symptom relief, and prevention of complications. However, the diverse clinical presentations, complex diagnostic criteria, and multifactorial considerations in treatment planning present considerable challenges for clinicians [6]. Endodontic emergencies can be particularly demanding for both clinicians and patients, requiring knowledge of how complications may arise, how to manage them predictably, and which preventive measures to implement [7]. As complications can affect outcomes differently, clinicians should assess prognostic factors and choose suitable treatment strategies [8]. In these high-stakes scenarios where rapid decision-making and stress management are crucial, AI-based models capable of generating accurate and comprehensive responses can serve as valuable support tools for practitioners.

Released in November 2022, ChatGPT (OpenAI, San Francisco, CA, USA) is a LLM capable of providing rapid and fluent responses while interacting with users in a human-like manner [9]. Similarly, Google’s Gemini model employs natural language processing capabilities to deliver information in the medical and dental fields [10,11]. DeepSeek-R1 (DeepSeek Inc., Hangzhou, China), launched in January 2025, is designed to address complex queries at a lower cost while offering advanced reasoning capabilities [12]. These models use large datasets and advanced algorithms to provide clear, personalized answers, improving health literacy and patient engagement [13,14]. However, their use in patient education carries certain limitations, including potential inaccuracies, biases in training data, and the inability to account for individual patient circumstances, necessitating careful implementation [15,16]. Evaluating these models comparatively is therefore crucial for ensuring their reliable and effective use in endodontic patient education.

Previous studies indicate that models such as ChatGPT can accurately answer multiple-choice questions derived from dental licensing examinations, demonstrating performance comparable to human dental graduates [17]. Moreover, several investigations suggest that LLMs may facilitate patients’ access to oral health information by providing clear and comprehensible responses [18,19]. However, the applicability of these findings to clinical patient education and health literacy remains largely unverified, highlighting the need for systematic, comparative evaluations of LLMs within dental educational contexts [20,21].

To our knowledge, LLMs have not yet been comparatively evaluated specifically in the context of patient education in dentistry. Existing studies focusing on endodontic questions suggest that LLMs can provide accurate, clear, and comprehensive answers, supporting informed decision-making and reducing patient anxiety [22,23,24,25]. Nonetheless, the clinical reliability of these responses has not been thoroughly validated, particularly in urgent or complex endodontic cases where timely understanding is critical for patient safety and decision-making. These gaps underscore the importance of a systematic evaluation of DeepSeek V3, GPT 5, and Gemini 2.5 Flash to determine their potential utility in endodontic patient education and health literacy.

The aim of this study was to assess the performance of DeepSeek V3, GPT-5, and Gemini 2.5 Flash in answering patients’ frequently asked questions (FAQs) about root canal therapy, focusing on accuracy and comprehensiveness. The study hypothesizes that DeepSeek V3, GPT 5, and Gemini 2.5 Flash differ in their ability to respond accurately and comprehensively. These models were selected to provide both comparative and practical insights: DeepSeek V3, released in 2025, is a recently developed model [26]; GPT 5 has been reported as widely used [27]; and Gemini 2.5 Flash reportedly employs a distinct algorithmic approach [28]. Selecting LLMs with these differing characteristics allows for the evaluation of potential performance differences.

## 2. Materials and Methods

### 2.1. Study Design

This study was conducted to compare the performance of three AI-based LLMs—DeepSeek V3, GPT 5, and Gemini 2.5 Flash—in responding to patients’ FAQs about root canal treatment. As the study did not involve human or animal subjects, ethical approval was not required.

### 2.2. Question Set

In this study, the most FAQs reflecting patients’ concerns about root canal treatment were identified. The questions were developed based on the informative content under the ‘*Root Canal Treatment*’ section of the official website of the American Association of Endodontists (AAE) (https://www.aae.org/patients/root-canal-treatment/; accessed on 10 September 2025) [29]. This ensures transparency and allows reproducibility of the question set.

Two experienced endodontists independently reviewed all FAQs for relevance and clarity, with a predefined inter-expert agreement threshold of ≥80%. Questions that did not meet this threshold were discussed and revised until consensus was achieved. The questions were categorized into eight main domains to reflect potential patient concerns: (1) general definition and purpose, (2) symptoms and indications, (3) during and after the procedure, (4) alternatives, success, and risks, (5) pain, safety, and misconceptions, (6) daily life, quality of life, and esthetics, (7) cost and insurance, and (8) specialty and referral. The questions were exclusively derived from patient educational content and did not cover technical or procedural steps. The categorization of FAQs into eight main domains was not intended for grouping purposes but rather aimed to facilitate a comprehensive understanding of patient concerns regarding all aspects of root canal treatment. The responses provided by the LLMs were systematically evaluated in terms of their accuracy, comprehensiveness, and contribution to patient health literacy.

A total of 37 FAQs were generated (Table 1), representing the most common concerns encountered by patients in both clinical practice and daily life. These were adapted from the publicly available educational content of the AAE into a standardized question format suitable for evaluation.

### 2.3. Response Generation and Data Collection

**Figure 1:**
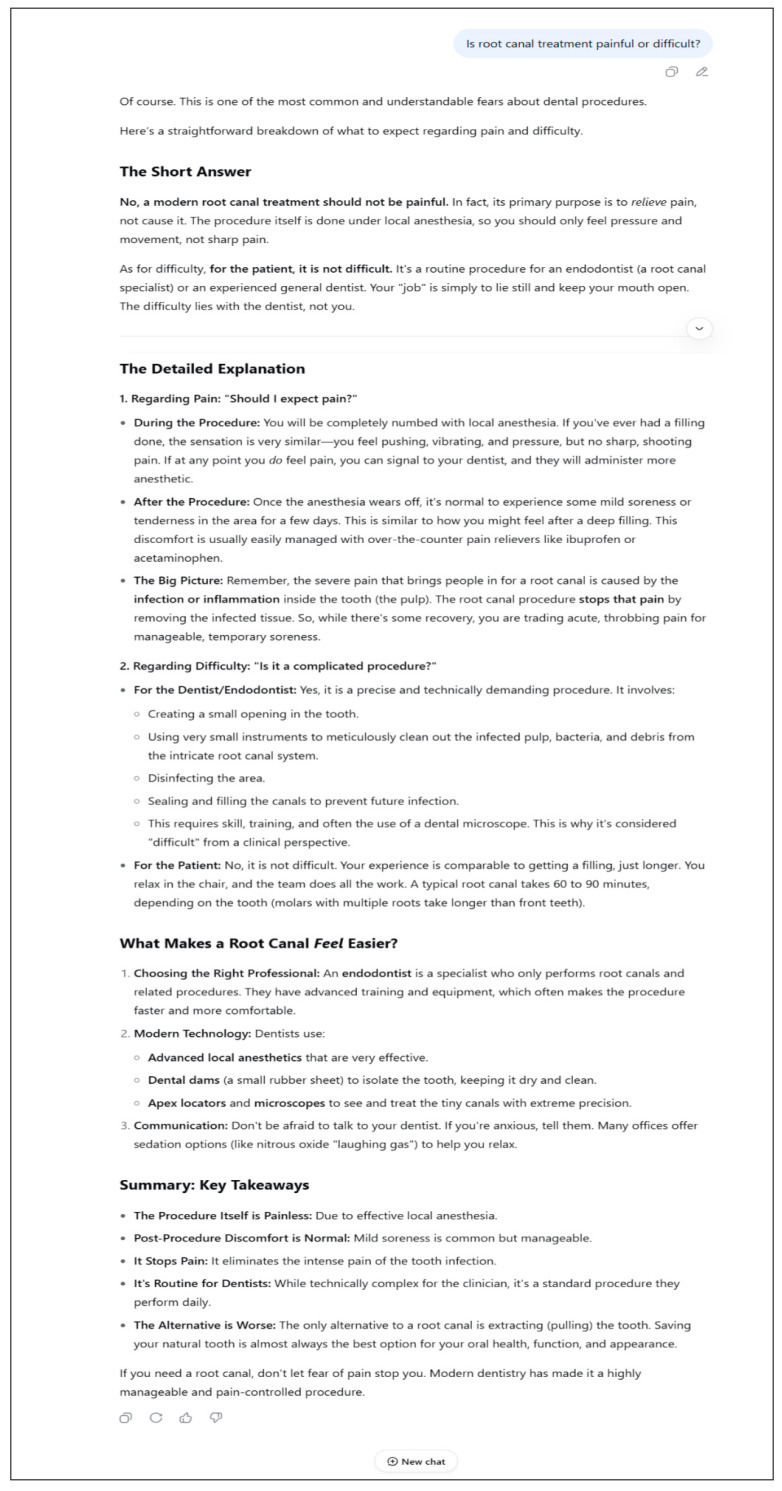
Sample answer from DeepSeek V3.

**Figure 2:**
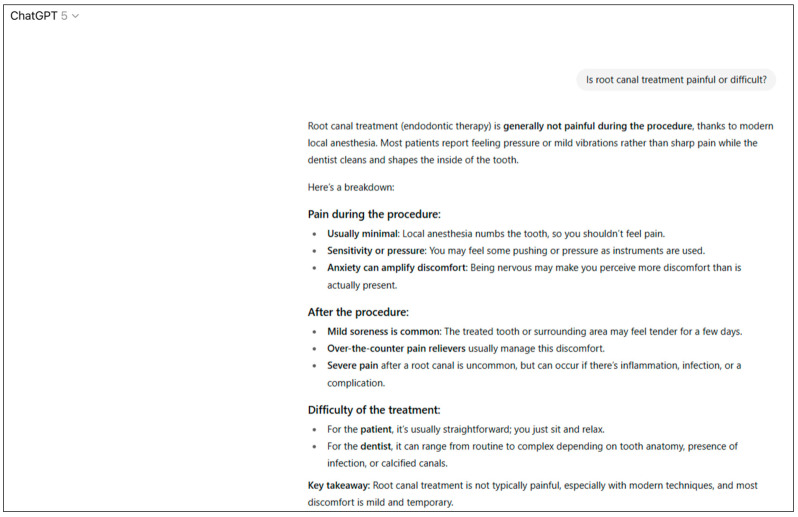
Sample answer from GPT 5.

**Figure 3:**
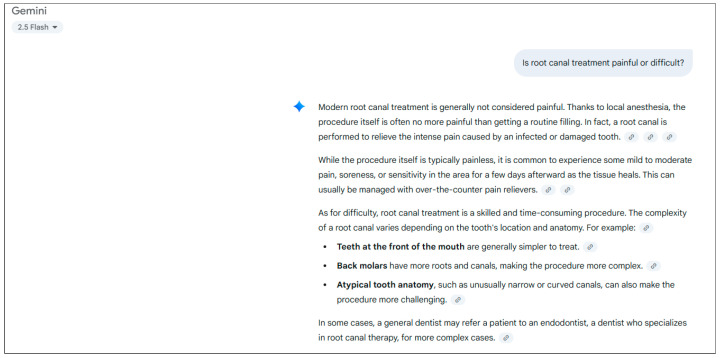
Sample answer from Gemini 2.5 Flash.

Each of the 37 questions was sequentially presented to DeepSeek V3, GPT 5, and Gemini 2.5 Flash. The questions were submitted in a standardized manner without additional prompts, clarifications, or rephrasing. Each response was recorded in its original form for evaluation. To ensure consistency, the session was reset or a new window was opened before presenting the next question. All procedures were conducted using the same computer (Asus TUF Gaming F15 FX507ZC4_FX507ZC4; ASUSTeK Computer Inc., Taipei, Taiwan), a 4.5G internet connection and a virtual private network (VPN) server (version 3.9; Astrill Systems Corp., Santa Clara, CA, USA). Examples of responses generated by DeepSeek V3, GPT 5, and Gemini 2.5 Flash for the question “*Is root canal treatment painful or difficult?*” are presented in Figure 1, Figure 2 and Figure 3, recorded exactly as received without any modifications or prompts.

For each question, responses were generated and recorded once by the LLMs. The test–retest procedure was conducted by rescoring the same 37 responses two weeks later, allowing assessment of rater consistency (rater stability). The responses were blinded so that the evaluators were unaware of which model produced them; for this purpose, all responses were transferred into a Word document (Microsoft Corporation, Redmond, WA, USA), and the LLM and stylistic cues were concealed.

All LLMs were accessed through their official web-based interfaces between July and August 2025. The models and access details were as follows:

- ChatGPT 5 (OpenAI Global, San Francisco, CA, USA; model identifier: gpt 5; access date: July 2025; access platform: local inference; account type: self-hosted; default generation settings: temperature = 1.0; top-p = 1.0; no context window limits were exceeded).
- DeepSeek V3 (DeepSeek, Hangzhou, China; model identifier: deepseek v3; access date: June 2025; access platform: local inference; account type: self-hosted; default generation settings: temperature = 1.0; top-p = 1.0; no context window limits were exceeded).
- Gemini 2.5 Flash (Google, Mountain View, CA, USA; model identifier: Gemini 2.5 Flash; access date: August 2025; access platform: local inference; account type: self-hosted; default generation settings: temperature = 1; top-p = 0.95; no context window limits were exceeded).

### 2.4. Evaluation Criteria and Scoring Method

Each response was evaluated according to two predefined criteria:

*Accuracy*: The degree to which the response reflected current scientific evidence and clinical practice, assessing the factual correctness, reliability, and consistency of the information with established endodontic principles and guidelines.

*Comprehensiveness*: The extent to which the response fully and systematically addressed all relevant aspects of the question, covering both clinical and theoretical dimensions, and evaluated how well the answer captured the scope, depth, and clarity necessary for patient understanding.

Two experienced dental specialists (B.D.C. and O.C.) independently scored the responses in a blinded manner. The evaluators were unaware of which model had generated each response; all texts were transferred into a Microsoft Word document (Microsoft Corporation, Redmond, WA, USA), and any stylistic or brand-related cues were removed prior to scoring. Before scoring, the evaluators participated in a structured calibration and anchoring session using example responses not included in the study. These examples, focusing on the procedural steps of root canal treatment, were used to standardize the consistent application of the scoring criteria.

Responses were rated separately for accuracy and comprehensiveness using a five-point Likert scale [9]. The scale was conceptually aligned with the DISCERN instrument, a validated tool for assessing the quality of consumer health information [30]. Specifically, the ‘reliability’ and ‘quality of information’ domains of DISCERN were used as theoretical anchors for the definitions of accuracy and comprehensiveness in this study. While the scale was adapted to the present design, it retained conceptual consistency with established patient-education quality constructs. In cases where the two evaluators’ scores differed by more than one point, consensus was reached through adjudication. This approach ensured conceptual validity and reproducibility of the evaluation process. The scoring criteria are presented in Table 2.

### 2.5. Data Recording and Management

All data were recorded in Microsoft Excel (Microsoft Corporation, Redmond, WA, USA). Each entry included the model, question number, raw response, and the corresponding scores for accuracy and comprehensiveness. Scores from the test–retest procedure were documented in separate columns.

### 2.6. Statistical Analysis

Statistical analyses were performed using IBM SPSS Statistics for Windows, Version 26.0 (IBM Corp., Armonk, NY, USA). As the data were ordinal (Likert-type), non-parametric tests were used; therefore, no assumption of normality was required. Differences between the three LLMs were analyzed using the Kruskal–Wallis H test, followed by pairwise comparisons with the Mann–Whitney U test. When significant results were found, pairwise comparisons were conducted using the Mann–Whitney U test with Bonferroni correction to adjust for multiple testing. Inter-rater agreement and test–retest reliability were assessed by rescoring the same responses after a two-week interval using the intraclass correlation coefficient (ICC) based on a two-way random-effects model with 95% confidence intervals to evaluate rater consistency. Descriptive statistics (mean, standard deviation, and median) were reported.

Effect sizes were calculated to quantify the magnitude of statistically significant differences. For the Kruskal–Wallis tests, eta-squared (η^2^) values were computed using the formula η^2^ = (H − k + 1)/(N − k), where H is the Kruskal–Wallis statistic, k represents the number of groups, and N is the total sample size. For pairwise comparisons using the Mann–Whitney U test, Cliff’s delta (δ) was calculated as a non-parametric measure of effect size, reflecting the degree of stochastic superiority between two groups. Effect sizes were interpreted according to conventional thresholds as small (η^2^ = 0.01 or δ = 0.147), medium (η^2^ = 0.06 or δ = 0.33), and large (η^2^ ≥ 0.14 or δ ≥ 0.474) [31]. This approach ensured a robust and interpretable evaluation of differences across models, accounting for the ordinal nature of Likert-scale data. A *p*-value < 0.05 was considered statistically significant.

## 3. Results

The responses generated by the three LLMs were independently evaluated by two experienced specialist dentists (B.D.C. and O.C.). Inter-rater agreement, assessed using a two-way random-effects ICC model for consistency, was excellent for both accuracy (ICC = 0.92, 95% CI: 0.88–0.95) and comprehensiveness (ICC = 0.91, 95% CI: 0.87–0.95). Test–retest reliability, evaluated by rescoring the same 37 responses after a two-week interval, demonstrated high consistency across all models, with ICC values of 0.90 (95% CI: 0.85–0.94) for accuracy and 0.89 (95% CI: 0.84–0.93) for comprehensiveness. These results confirm that the evaluation process was robust, reproducible, and consistent.

A total of 37 FAQs about root canal treatment were scored for each model. DeepSeek V3 achieved the highest scores, with a mean accuracy of 4.81 ± 0.39 (median 5) and mean comprehensiveness of 4.78 ± 0.41 (median 5). GPT 5 showed moderate performance, scoring 4.0 ± 0.0 (median 4) for accuracy and 4.05 ± 0.4 (median 4) for comprehensiveness. Gemini 2.5 Flash obtained the lowest scores, with 3.83 ± 0.68 (median 4) for accuracy and 3.81 ± 0.7 (median 4) for comprehensiveness.

Statistical analysis using the Kruskal–Wallis test revealed significant differences among the models for both accuracy (*p* < 0.001) and comprehensiveness (*p* = 0.001). Pairwise comparisons indicated that DeepSeek V3 significantly outperformed both GPT 5 and Gemini 2.5 Flash (*p* < 0.05), whereas no significant difference was observed between GPT 5 and Gemini 2.5 Flash (*p* > 0.05). The statistical analysis results regarding the performance of the three LLMs in terms of accuracy and comprehensiveness are presented in Table 3.

Pairwise comparisons of the LLMs in terms of accuracy and comprehensiveness were conducted using the Mann–Whitney U test (Table 4). The overall Kruskal–Wallis test results showed η^2^ = 0.49 for accuracy and η^2^ = 0.39 for comprehensiveness, indicating moderate-to-large statistically significant differences among the LLMs.

For accuracy:

- DeepSeek V3 versus GPT 5 showed *p* < 0.001 and a Cliff’s delta (δ) of 0.81, indicating a very large and statistically significant difference.
- DeepSeek V3 versus Gemini 2.5 Flash yielded *p* < 0.001 and δ = 0.71, reflecting a large and significant difference.
- GPT 5 versus Gemini 2.5 Flash had *p* = 0.109 and δ = 0.16, which was not statistically significant and represents a small effect.

For comprehensiveness:

- DeepSeek V3 versus GPT 5 showed *p* < 0.001 and δ = 0.69, indicating a highly significant difference.
- DeepSeek V3 versus Gemini 2.5 Flash had *p* < 0.001 and δ = 0.70, again reflecting a large and statistically significant difference.
- GPT 5 versus Gemini 2.5 Flash yielded *p* = 0.058 and δ = 0.21, which was not statistically significant, although it indicates a small-to-moderate effect potential.

These results demonstrate that DeepSeek V3 performed statistically superior to the other models in both accuracy and comprehensiveness. No significant differences were observed between GPT 5 and Gemini 2.5 Flash for either criterion.

**Figure 4:**
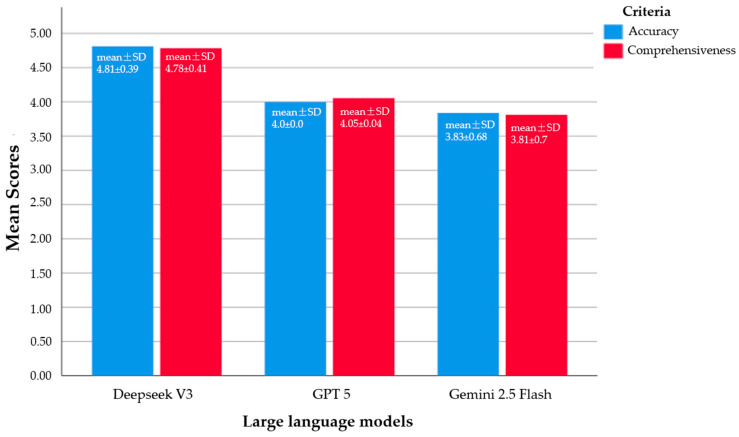
A clustered bar chart illustrating the mean and standard deviation (SD) values of the FAQs for each LLM, evaluating the Likert scores in terms of accuracy and comprehensiveness.

Figure 4 illustrates the mean accuracy and comprehensiveness scores of the three models. DeepSeek V3 consistently achieved the highest scores (mean ± SD: 4.81 ± 0.39 for accuracy, 4.78 ± 0.41 for comprehensiveness), GPT 5 performed moderately (4.0 ± 0.0 for accuracy, 4.05 ± 0.4 for comprehensiveness), and Gemini 2.5 Flash scored the lowest (3.83 ± 0.68 for accuracy, 3.81 ± 0.7 for comprehensiveness), highlighting significant differences in the overall quality of responses provided by each LLM.

**Figure 5:**
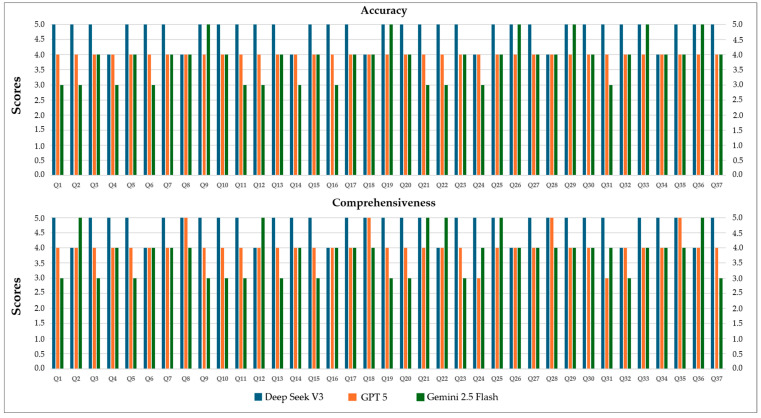
Accuracy and comprehensiveness scores of three LLMs for 37 root canal treatment questions (Q: question).

Figure 5 illustrates the accuracy and comprehensiveness scores for each of the 37 FAQs about root canal treatment by patients, evaluated across the DeepSeek V3, GPT 5, and Gemini 2.5 Flash models. DeepSeek V3 achieved the highest scores (median 5) for both criteria, while GPT 5 and Gemini 2.5 Flash exhibited lower and similar scores (median 4). The variation in scores was particularly noticeable for Gemini 2.5 Flash. These visual findings are consistent with previous statistical analyses and support the superior performance of DeepSeek V3 compared to the other models (Kruskal–Wallis, *p* < 0.001 for accuracy, *p* = 0.001 for comprehensiveness).

## 4. Discussion

Interest in LLMs has been growing in dentistry, as in many other fields. Some studies have examined the ability of LLMs to answer multiple-choice questions, while others have explored their capacity to respond to open-ended questions across various clinical scenarios [20,21,32,33]. The level of success varies depending on the specific LLM used, the clinical domain, and the type of question. One of the most important considerations when using chatbots as a source of medical information is the formulation of questions, which can substantially influence the quality of responses [34]. Open-ended questions are particularly valuable because they capture the nuances of medical decision-making more effectively [35]. Conversational LLMs have also demonstrated strong performance in summarizing health-related texts, answering general medical questions, and collecting patient information [36].

This study evaluated the performance of DeepSeek V3, GPT 5, and Gemini 2.5 Flash in responding to FAQs about endodontic root canal treatment, focusing on accuracy and comprehensiveness. The results demonstrated clear differences among the models. DeepSeek V3 achieved the highest scores in both accuracy (4.81 ± 0.39) and comprehensiveness (4.78 ± 0.41), showing statistically significant superior performance compared to GPT 5 and Gemini 2.5 Flash (*p* < 0.05). GPT 5 exhibited moderate performance, while Gemini 2.5 Flash received the lowest scores. These findings confirm our hypothesis that the models differ in their ability to respond accurately and comprehensively. The observed differences in performance might reflect variations in training datasets, algorithmic architectures, and model capacities, which may in turn affect the quality of information and patient education outcomes [1,4,37].

In our study, 37 open-ended questions compiled from AAE patient education materials and reliable online resources were presented to the LLMs only once, without rephrasing, in a manner designed to simulate a genuine clinical consultation. The responses were evaluated independently and in a blinded fashion by two experienced dentists; the identity of the generating model was concealed, and a brief calibration session was conducted prior to assessment. Our findings revealed that DeepSeek V3 demonstrated high proficiency in understanding the questions and generating contextually appropriate responses, whereas GPT 5 and Gemini 2.5 Flash exhibited lower accuracy and comprehensiveness scores. Similarly, Mago et al. [38] reported that although GPT 3 can assist oral radiologists in diagnosing pathologies, it should not be relied upon as a primary diagnostic reference. Likewise, in the diagnostic accuracy study by Bragazzi et al. [39], ChatGPT was shown to misclassify superimpositions as carious lesions and to be inadequate in detecting bone loss associated with periodontal disease. Such shortcomings carry the potential to mislead patients, underscoring the necessity of critically evaluating LLM-based informational sources within the context of health literacy and patient education.

Our findings suggest that LLMs could greatly benefit patient education and information provision. The superior performance of DeepSeek V3 might be associated with its advanced reasoning capabilities and extensive training dataset [12,20], but this remains speculative. Although GPT 5 achieved higher performance scores than Gemini 2.5 Flash, the difference was not statistically significant. Additionally, responses from both models were more limited and generalized compared to DeepSeek V3. Similarly, Aljamani et al. [37] reported that ChatGPT 3.5 outperformed Gemini in responding to questions about endodontic pain in terms of quality and reliability.

Evidence from studies investigating LLM applications in healthcare indicates that these models may offer considerable advantages for clinical practice and patient education. The study by Zhang et al. [24] compared the response comprehensiveness of ChatGPT 3.5 and ChatGPT 4 across different language contexts and question types in gingival and endodontic health, highlighting the decisive role of model architecture and training processes on performance. The authors [24] reported that while LLMs provide high accuracy and comprehensiveness for general knowledge questions, their performance may decline for questions requiring specialized expertise; this observation parallels the differences noted in our comparison of DeepSeek V3, GPT 5, and Gemini 2.5 Flash. The superior performance of DeepSeek V3 can be associated, as Zhang et al. [24] noted for ChatGPT 4, with the advantages conferred by a larger and optimized training dataset and more advanced model architecture [24,40,41].

Recent studies have shown that the factors influencing LLM performance are not limited to model capacity alone, but also encompass more complex dimensions such as linguistic diversity, contextual nuances, and the interplay of multimodal content [24,42,43]. Higher performance has been reported for English questions, whereas Chinese responses have been more limited, likely due to differences in training data distribution and terminology [24]. Similarly, in our study, the high accuracy and comprehensiveness scores of DeepSeek V3 can be attributed to its English-focused dataset and optimized architecture. In contrast, the limited responses from GPT 5 and Gemini 2.5 Flash highlight the need for careful evaluation of LLM outputs. This aspect is particularly important for improving health literacy, as information generated by LLMs must be reliable, understandable, and clinically consistent to enable patients to correctly interpret their treatment options and make informed decisions. These findings emphasize the importance of model selection, dataset coverage, and the clinical complexity of questions for the reliable use of LLMs in patient education and clinical decision support.

Emerging evidence on the clinical decision support capabilities of LLMs in endodontics suggests that these systems demonstrate variable performance with respect to accuracy and comprehensiveness [9,20,32]. Özbay et al. [9] compared the responses of Google Bard, ChatGPT 3.5, and ChatGPT 4 to endodontic clinical questions and reported that ChatGPT 4 achieved the highest accuracy and information coverage with a lower misinformation rate than the other models. Similarly, our study found that DeepSeek V3 had higher accuracy and comprehensiveness scores than GPT 5 and Gemini 2.5 Flash. This result underscores the importance of advanced training datasets and optimized model architecture in improving response quality [12,20]. However, the literature frequently highlights ethical and safety concerns related to potential misinformation, despite the promise of LLMs in clinical decision support and patient education [36,44]. Even with high accuracy and comprehensiveness, patients should exercise caution, especially in complex clinical scenarios. Models should not be used as primary sources without human expert oversight [9,30,38]. The limited, general responses of GPT 5 and Gemini 2.5 Flash in our study underscore the importance of selecting the right model, ensuring high-quality datasets, and designing effective questions for reliable LLM use [20,32].

Using LLMs for patient education has significant advantages, but it also has limitations. LLMs may not always provide accurate or complete responses to complex or individualized clinical scenarios, nor do they have direct access to literature updated beyond their existing training datasets [9,36]. Therefore, responses generated by LLMs should be considered solely as supportive and informative tools, and expert clinician oversight remains essential [18,30].

The limitations of our study include the evaluation of only specific questions reflecting patient concerns related to root canal treatment, the exclusion of questions associated with other clinical applications (such as patient inquiries concerning endodontic-periodontic, endodontic-surgical, or endodontic-orthodontic issues), and the inability to directly verify the clinical accuracy of the model responses. Moreover, the dataset was limited to English-language questions, which may constrain the generalizability of the findings to multilingual or culturally diverse patient populations. Another limitation is that the five-point Likert scale used in this study was adapted from the DISCERN instrument but was not independently validated, which may limit the external reproducibility of the scoring process. Despite blinded scoring and standardized calibration, some degree of evaluator subjectivity may have persisted, which should be considered when interpreting the results. Additionally, although the evaluators were experienced dental specialists, having the scoring conducted by only two raters may limit the robustness and statistical power of the results. This situation underscores the need for study designs that include a larger number of evaluators to ensure greater statistical reliability.

Future studies should focus on testing LLMs across diverse clinical scenarios and patient demographics, enabling continuous monitoring and improvement of model performance. In this context, advanced LLMs such as DeepSeek V3, when properly trained and supervised, demonstrate the potential to provide accurate and comprehensive responses to patients’ FAQs about root canal treatment. However, to more comprehensively evaluate the relative performance of LLMs, studies should be planned that include comparative analyses with other LLMs. Additionally, to further strengthen reliability and enable more rigorous comparisons that include robust variance estimates, a larger number of independent endodontic specialists should be involved. Moreover, guidance from dental associations and adherence to scientific standards may play a critical role in ensuring the safe and effective integration of these AI-based technologies into health literacy and patient education [4,44,45,46].

## 5. Conclusions

Within the limitations of this study, the following conclusions were drawn:

- LLMs showed significant differences in accuracy and comprehensiveness when responding to patient FAQs regarding root canal treatment.
- DeepSeek V3 achieved relatively higher scores in accuracy and comprehensiveness compared to GPT 5 and Gemini 2.5 Flash, although these findings are based on a Likert-based rubric and a limited English-language dataset, and should not be interpreted as definitive evidence of absolute superiority.
- Advanced LLMs may serve as supportive tools in patient education and health literacy related to root canal treatment; however, expert clinician oversight remains essential in clinical decision-making and treatment planning.
- When appropriately utilized, LLMs can contribute to enhancing patient awareness and supporting satisfaction during endodontic treatment processes.

## Figures and Tables

**Table 1:** Frequently asked questions by patients about root canal treatment.

**General definition and purpose**
1. What is a root canal treatment?
2. Why is root canal treatment needed?
3. How does root canal treatment save a tooth?
4. Does root canal treatment cause the tooth to die?
**Symptoms and indications**
5. How can I know if I need a root canal treatment, and what are the main signs?
6. Can I still need a root canal treatment even if I have no pain?
7. Can all teeth be treated with a root canal treatment, or are there situations where it cannot be performed?
**During and after the procedure**
8. Is root canal treatment painful or difficult?
9. How many visits are usually required for a root canal treatment?
10. How is a root canal performed step by step?
11. What is the recovery process like after root canal treatment?
12. Will my tooth function normally after the treatment?
13. When can I eat after a root canal?
14. Is special care needed for my tooth after the treatment?
15. Will my tooth turn dark after the root canal treatment?
16. Will I need a crown after root canal treatment?
**Alternatives, success, and risks**
17. Are there any alternatives to root canal treatment?
18. What is the success rate of root canal treatment?
19. Can a root canal treatment fail?
20. What happens if I do not get a root canal treatment?
21. Is a root canal treatment or tooth extraction better?
22. What happens if a previously root canal treated tooth has problems again?
**Pain, safety, and misconceptions**
23. Can root canal treatment be harmful to overall health?
24. Are the roots of the tooth removed during a root canal treatment?
25. Can I be sedated or put to sleep for a root canal treatment?
**Daily life, quality of life, and esthetics**
26. Can I return to work or school immediately after a root canal treatment?
27. Can I smoke after a root canal treatment?
28. Can I drink alcohol after the procedure?
29. Can I drive after a root canal treatment?
30. How will my tooth look aesthetically after root canal treatment?
**Cost and insurance**
31. How much does root canal treatment cost?
32. How does the cost of a root canal compare to tooth extraction?
33. Is root canal treatment covered by dental insurance?
**Specialty and referral**
34. Which dentists can perform root canal treatment?
35. What is the difference between a general dentist and an endodontist?
36. Are endodontists available for emergency treatment on weekends?
37. In which cases should I definitely see an endodontist for a root canal?

**Table 2:** Scoring Criteria for Accuracy and Comprehensiveness.

	Accuracy		Comprehensiveness	
Score	Category	Definition	Category	Definition
1	Completely incorrect	Inconsistent with current scientific evidence or clinically misleading	Not adequate	Addresses only a minor part of the question; highly inadequate explanation
2	More incorrect than correct	Includes some correct elements but overall misleading	Somewhat adequate	Covers the question partially; omits key points
3	Partially correct/partially incomplete	Contains essential information but with noticeable gaps or superficial detail	Adequate	Provides a general answer to the question; lacks depth in some areas
4	More correct than incorrect	Minor factual or terminological inaccuracies may exist	Very adequate	Addresses most aspects of the question with sufficient and detailed explanation
5	Completely correct	Fully consistent with current evidence and clinical guidelines	Extremely adequate	Thoroughly and clearly covers all relevant aspects of the question in a balanced and informative manner

**Table 3:** Comparison of model performance scores.

Criteria	Large Language Models			*p*
	DeepSeek V3 ^a^	GPT 5 ^b^	Gemini 2.5 Flash ^c^	
	Mean ± SD; (Median) [Q1–Q3]	Mean ± SD; (Median) [Q1–Q3]	Mean ± SD; (Median) [Q1–Q3]	
Accuracy	4.81 ± 0.39; (5) ^b,c^ [5–5]	4 ± 0; (4) [4–4]	3.83 ± 0.68; (4) [3–4]	<0.001 *^,K^
Comprehensiveness	4.78 ± 0.41; (5) ^b,c^ [5–5]	4.05 ± 0.4; (4) [4–4]	3.81 ± 0.7; (4) [3–4]	0.001 *^,K^

^K^: Kruskal–Wallis test (post hoc pairwise comparisons were performed using the Mann–Whitney U test with Bonferroni correction), SD: Standard deviation, *p*: significance value, *: *p* < 0.05 ^a^: Difference with DeepSeek V3 *p* < 0.05, ^b^: Difference with GPT 5 *p* < 0.05, ^c^: Difference with Gemini 2.5 Flash *p* < 0.05.

**Table 4:** Effect sizes and pairwise comparisons for accuracy and comprehensiveness.

Criteria	Eta Squared (η^2^)	Pairwise Comparison	*p*	Cliff’s Delta (*δ*)
Accuracy	0.49	DeepSeek V3 vs. GPT 5	<0.001 *	0.81
		DeepSeek V3 vs. Gemini 2.5 Flash	<0.001 *	0.71
		GPT 5 vs. Gemini 2.5 Flash	0.109	0.16
Comprehensiveness	0.39	DeepSeek V3 vs. GPT 5	<0.001 *	0.69
		DeepSeek V3 vs. Gemini 2.5 Flash	<0.001 *	0.70
		GPT 5 vs. Gemini 2.5 Flash	0.058	0.21

η^2^: effect size for Kruskal–Wallis tests, Cliff’s delta (δ): effect size for pairwise Mann–Whitney comparisons, *p*: significance value, *: *p* < 0.05.

## Data Availability

The data that support the findings of this study are available from the corresponding author upon reasonable request.

## Abbreviations

The following abbreviations are used in this manuscript:

AI	Artificial Intelligence
LLM	Large Language Model
AAE	American Association of Endodontists
FAQ	Frequently Asked Questions
ICC	Intraclass Correlation Coefficient
SD	Standard Deviation
